# Effects of frequency and amount of stover mulching on soil nitrogen and the microbial functional guilds of the endosphere and rhizosphere

**DOI:** 10.3389/fmicb.2022.976154

**Published:** 2022-08-24

**Authors:** Wenchen Song, Jing Wang, Lei Hou

**Affiliations:** ^1^Key Laboratory of Ecology and Environment in Minority Areas, Minzu University of China, National Ethnic Affairs Commission, Beijing, China; ^2^College of Life and Environmental Sciences, Minzu University of China, Beijing, China; ^3^Beijing Pollution Source Related Affair Management Center, Beijing, China

**Keywords:** stover mulching, soil nitrogen, bacterial functional guild, fungal functional guild, root-soil interaction

## Abstract

Stover mulching as a conservation and sustainable agricultural practice is beneficial for maintaining soil nitrogen (N) requirements and plant health. The microbial functional guilds of the root and rhizosphere are important factors in the soil nitrogen cycle. However, it is unclear how the frequency and amount of stover mulching influence microbial functional guilds in the root and rhizosphere. Therefore, we investigated the responses of the microbial functional guilds in the endosphere and rhizosphere to maize stover mulching amounts (0, 1/3, 2/3, and total stover mulching every year) and frequencies (once every 3 years and twice every 3 years) under 10-year no-till management. The bacterial functional guilds of nitrogen fixation, nitrification, and anaerobic nitrate oxidation displayed the significantly correlation with C/N, total nitrogen, NO_3_^−^, and NH_4_^+^. The fungal functional guilds of plant pathogens and saprotrophs showed significantly correlations with C/N, total nitrogen, and NO_3_^−^. Moreover, we found that bacterial guilds play a pivotal role in maintaining N requirements at the jointing stage, whereas root endophytic fungal guilds play a more important role than bacterial guilds in regulating plant health at the mature stage. The frequency and amount of stover mulching had significant effects on the microbial functional guilds in the root and rhizosphere. Our data suggest that stover mulch application twice every 3 years is the optimal mulching frequency because it yielded the lowest abundance of nitrifying and anaerobic nitrate-oxidising bacteria and the highest abundance of nitrogen-fixing bacteria at the jointing stage, as well as the lowest abundance of fungal plant pathogens in roots at the mature stage.

## Introduction

Maize (*Zea mays* L.) is one of the three major food crops in the world, and China accounts for 23% of the global maize production (Wang et al., 2019a). As a well-known agricultural country, China is rich in crop stover resources. Stover is an important organic fertiliser resource, and returning it directly or indirectly to the soil will not only establish a healthy soil ecosystem, but it can also be an effective measure to improve land productivity (Li et al., 2018; Gao et al., 2019; Battaglia et al., 2021; Thakur and Kumar, 2021). However, due to the lack of rural labour force, high treatment costs, and farmers’ lack of scientific knowledge, stovers are burned in a large area, polluting the air and wasting biological nutrition (Ren et al., 2019; Wu et al., 2020). According to the traditional farming system in Northeast China, most crop stovers are burned in the field after harvest each year (Wang et al., 2019b). Soil nitrogen (N) levels and availability are key determinants of maize yield, N accumulation, and grain protein content (Xing et al., 2019). Long-term stover return was beneficial to the accumulation of soil total N and available N (Wang et al., 2018, 2019c; Li et al., 2021). However, which tillage method of stover mulching is most conducive to crop growth and soil nitrogen accumulation is still unclear.

Stover mulching provides additional nutrients (i.e., carbon and nitrogen) to crops and soil microbial systems through decomposition (Maarastawi et al., 2019). The material and energy channels of bacteria and fungi are the main nitrogen migration and transformation pathways in soils of stover mulching systems (Li et al., 2021). Generally, bacteria always use easily available nutrient resources and generate rapid nutrient cycling in the soil, whereas fungi typically decompose more difficult nutrients with slower cycling (Thakur and Geisen, 2019). Stover mulching significantly affected the composition and function of soil biological communities because of the C/N ratio of stover and the decomposition rate under different tillage management, thereby affecting the accumulation of soil nitrogen (Li et al., 2018; Martínez-García et al., 2018; Kou et al., 2020). A study showed that the decomposition efficiency of the primary decomposers and the composition of soil biological communities were both affected by the amount and frequency of stover cover (Kou et al., 2020; Wang et al., 2020a,b). The proper amount of stover mulching can increase the number of bacteria, whereas excessive mulching could inhibit the function of the microbial community (Zhang et al., 2015). A previous study revealed that No-tillage with stover mulching significantly affected soil fungal abundance, but not bacterial diversity (Wang et al., 2020b). The other found that it was the frequency of stover mulching rather than the amount affected the decomposition and food web of microorganisms in soils (Kou et al., 2020). Therefore, we speculate that the amount and frequency of stover application will affect the soil microbial community and consequently the nitrogen nutrition status of the soil. However, there were still only few studies on the mechanism of soil microbial functional guilds in soil N accumulation under stover mulching.

The productivity of the aboveground parts is mainly affected by the root-associated microbiota (de Vries et al., 2017; Fitzpatrick et al., 2018). Rhizo-microorganisms affect the productivity and sustainability of agroecosystems by regulating nutrient cycling, changing soil structure, and inhibiting plant diseases (Pathan et al., 2015; Hassan et al., 2019; Olanrewaju et al., 2019). Previous studies mainly focused on the effects of different tillage practices (traditional tillage, no tillage, and organic tillage) on soil microbial composition and nutrient content (Lupatini et al., 2016; Martínez-García et al., 2018; Schmidt et al., 2018). At present, there are few reports on the effects of tillage on root endophytic and rhizosphere microorganisms that affect soil N (Dahlstrom et al., 2020). Plants can transport organic matter high in C/N ratio from the shoot into the underground pools, which stimulates rhizosphere microorganisms to decompose organic matter and produce N (Pausch and Kuzyakov, 2018). Thus, plants not only obtain N, but also change the N content and composition of soil. Similar to root exudates, stover is also an organic matter with a high C/N ratio (Li et al., 2021). The decomposition process of stover also changes the composition of the microorganisms and N content in soil (Rajkovich et al., 2012; Ghafoor et al., 2017; Li et al., 2020). However, whether stover mulching could affected the microbial functional guilds in the endosphere and rhizosphere, and then affect soil N? This problem is still unclear.

In this study, we focused on the effect of different frequencies and amounts of stover mulching on soil N and microbial functional guilds of the root and rhizosphere. The study aimed to determine the optimal frequency and amount of stover mulching that will maintain soil N requirements and the health of root endophytic and rhizosphere microbial communities. This study will be of great ecological significance in protecting and promoting agricultural production.

## Materials and methods

### Experimental design

Field experiments were conducted using maize stover in a maize cropping system at a site located in Aohan, Inner Mongolia (42.26′N, 119.70′E). The field experiment was established in the spring of 2010 in Aohan, Inner Mongolia. The climate was monsoon, continental climate, and semi-arid climate. The annual average temperature and precipitation were 6°C and 384 mm, respectively. The rainfall from June to September accounted for more than 70% of the total rainfall. This soil was classified as clay loam.

This land has been cultivated for 10 years. The experiment has performed 9 years and it was randomly plotted using six treatments: FQ0 (control with no maize stover return), Q1/3 (1/3 stover mulching per year), Q2/3 (2/3 stover mulching per year), FQ1 (100% stover mulching per year), F1/3 (stover mulching in the first year of a three-year cycle), and F2/3 (stover mulching in the first 2 years of a three-year cycle). The maximum application amount (total stover mulch; FQ1) was approximately 7.5 t/ha. The 30 cm high stubbles were left after harvest and the remaining plant parts were crushed, then applied to the soil surface at rates appropriate for the experimental treatments. Each treatment was replicated in four plots, and the area of each plot was 8.7 m × 30 m. The maize stover was used for mulching experiments, and the compound fertiliser (N-P_2_O_5_-K_2_O, 26–12%) was applied before maize sowing at 900 kg/ha.

### Field sampling and laboratory analysis

In brief, continuous soil sampling was conducted during the jointing (May to June), flowering (July), and mature (September) stages of maize in 2020. In each plot, five maize plants and their roots were randomly selected to collect their roots as samples. Diameter less than 2 mm fine root samples were selected and loaded into a 2 ml sterile tube filled with sterile water. The rhizosphere soil that cohered to the roots was separated by 15 min shaking and 10,000 r/min centrifuging in sterile tubes. Then, the fine roots were removed and rinsed with deionized sterile water. Finally, rhizosphere soil and roots were stored at −80°C for subsequent analysis. For soil chemical analysis, an auger was used to collect bulk soil samples from undisturbed soil, sieved into a 2 mm mesh, and stored at 4°C. The wet oxidation (K_2_Cr_2_O_7_) method was used to measure the soil organic carbon. Total soil nitrogen, NH_4_^+^-N, and NO_3_^−^N were extracted according to the method described by Kou et al. (2020).

### DNA extraction

The DNA extraction were performed as described by Song et al., 2020. DNA (0.5 g) was extracted from rhizosphere soil and root samples using the MO BIO’s PowerSoil DNA isolation kit (Qiagen, Germany) according to the manufacturer’s protocol. The maize roots were ground in liquid nitrogen prior to DNA extraction. A NanoDrop 2000 spectrophotometer (Waltham, Massachusetts, United States) was used to assess the concentration of the extracted DNA. The bacterial and fungal communities were investigated by targeted amplification of the V4 region of the bacterial 16S rRNA and the fungal ITS region, respectively. The selected regions were amplified using universal primers 515F (GTGCCAGCMGCCGCGGTAA) and 806R (GGACTACHVGGGTWTCTAAT; for bacterial 16S rRNA) and 5.8SFun (AACTTTYRRCAAYGGATCWCT) and ITS4Fun (AGCCTCCGCTTATTGATATGCTTAART; for fungal ITS region). The PCR mix included 1 μl DNA, 2.5 μl forward and reverse primers, and 5 μl PCR buffer. PCR was performed as follows: denaturation for 3 min at 95°C; followed by 27 cycles of 95°C for 30 s, 55°C for 30 s, 72°C for 45 s; and a final extension at 72°C for 10 min. The PCR reaction procedures and product purification were performed as described by Taş et al. (2014). The amplicon libraries were sequenced using the Illumina MiSeq platform (Illumina, USA) with a paired-end sequencing strategy.

### Bioinformatic analysis

The raw data were first screened and sequences were removed from consideration if they were shorter than 230 bp, had a low-quality score (≤ 20), contained ambiguous bases, or did not exactly match to primer sequences and barcode tags’ and separated using the sample-specific barcode sequences. The sequences obtained from the sequencer were processed using the QIIME2 pipeline (Caporaso et al., 2010). After removing barcodes and primers, quality control was performed by removing ambiguous reads and low-quality sequences. Paired ends of 16S rRNA and ITS reads were merged using FLASH tool (Magoc and Salzberg, 2011). After the removal of chimeric sequences, qualified sequences were clustered into operational taxonomic units (OTUs) using UPARSE based on 97% similarity. Taxonomic assignments of the representative sequence for each bacterial and fungal OTU were searched using the SILVA 138 SSU Ref NR99 and UNITE 8.2 databases (Nilsson et al., 2018). The complete dataset was submitted to the NCBI Sequence Read Archive (SRA) database under the accession number PRJNA758631.

### Statistical analyses

R (4.0.2) platform were used for statistical analysis, R packages of “ggplot2” (3.3.6) were used for data visualization. The significance of microbial abundance among treatments was evaluated using ANOVA with aov () function in the package of stats (version 4.0.2). The relationships between the predicted microbial function and soil chemical properties were analysed by Spearman’s correlation with corr.test () function in the package of stats (version 4.0.2). The Shannon index of microbial communities was calculated using the *Vegan* package (2.5-7). Because our data is nonlinear, the canonical correspondence analysis (CCA) for bacterial and fungal beta diversity in different times and tillage was performed using *Ape* package (5.6-2). Histogram analysis of species composition was performed, and a correlation heat map was drawn using the R packages *psych* (2.2.5) and *pheatmap* (1.0). A Tukey’s HSD test was used if the difference between groups was significant. FAPROTAX and FUNGuild were used to divide the functions of bacterial and fungal, respectively (Louca et al., 2016; Nguyen et al., 2016).

## Results

### Effects of different times and tillage on microbial taxa

**Figure 1:**
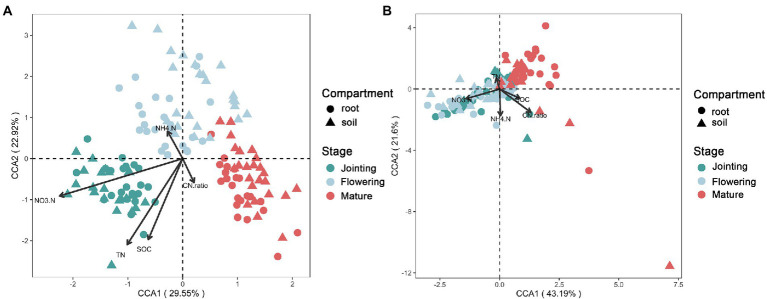
Canonical correspondence analysis of the **(A)** bacterial and **(B)** fungal beta-diversity and soil properties at three growth stages of maize.

Different growth stages of crops had effects on bacterial communities. CCA1 and CCA2 could explain 29.55 and 22.92% of the bacterial extraction sorting axis, respectively. Nitrate (NO_3_^−^), total nitrogen (TN), and soil organic carbon (SOC) were mainly determined by the bacterial community at the jointing stage, and ammonia (NH_4_^+^) was mainly determined by the bacterial community at the flowering stage (Figure 1A). The C/N ratio was related by the bacterial community at the mature stage (Figure 1A). For fungi, CCA1 and CCA2 could explain 43.19 and 21.60% of the extraction sorting axis, respectively. The fungal community structure at the flowering and jointing stages significantly affected NO_3_^−^, but had no significant effect at the mature stage (Figure 1B). The distribution of microbial community structure at different growth stages had a significant impact on crop growth stages (Figure 1).

**Figure 2:**
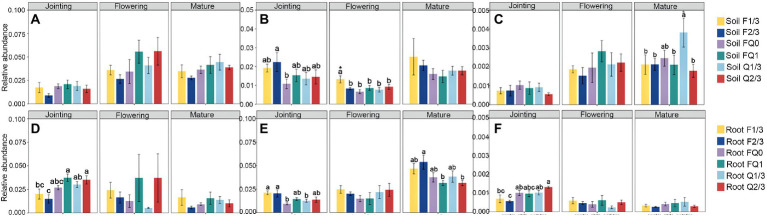
The relative abundance of functional guilds related to **(A)** nitrification **(B)** nitrogen fixation, and **(C)** anaerobic nitrate oxidation in rhizosphere soil; and the relative abundance of functional guilds related to **(D)** nitrification **(E)** nitrogen fixation and **(F)** anaerobic nitrate oxidation in roots. Different letters indicate significant differences at *p* < 0.05. * indicates significant differences at *p* < 0.01. No letter indicates significant differences at *p* > 0.05 between all groups. FQ0: control with no maize stover return; Q1/3: 1/3 stover mulch applied every year; Q2/3: 2/3 stover mulch applied every year; FQ1: 100% stover mulching every year; F1/3: stover mulching in the first year of each three-year cycle; F2/3: stover mulching in the first two years of each three-year cycle.

**Figure 3:**
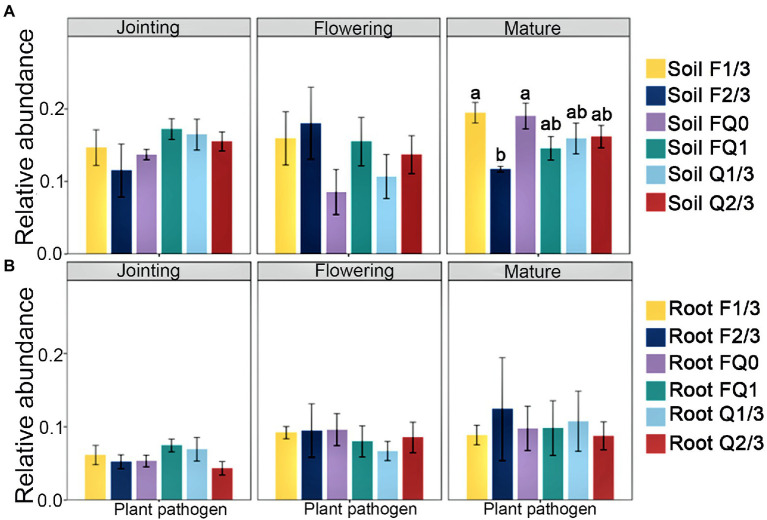
The relative abundance of plant pathogens in **(A)** rhizosphere soil and **(B)** roots. Different letters indicate significant differences at *p* < 0.05. No letter indicates significant differences at *p* > 0.05 between all groups. Treatments are defined in Figure 2.

At the jointing stage, the root nitrification bacterial abundance in FQ1 and Q2/3 was obviously higher than that in other groups, whereas F2/3 was the lowest among all groups (Figures 2A,D). At the flowering stage, the abundance of nitrogen fixation functional groups in the rhizosphere soil of F2/3 was significantly higher than that of FQ0, whereas F1/3 at the jointing stage was the highest among all groups (Figures 2B,E). The bacterial abundance of nitrogen fixation in the roots of FQ0 at the jointing stage was the lowest, whereas F2/3 at the mature stage was the highest among all groups (Figures 2B,E). The abundance of anaerobic nitrate oxidizing functional groups in the rhizosphere soil at the mature, flowering, and jointing stages were highest among all groups (Figures 2C,F). The abundance of fungal pathogens in the rhizosphere soil of F1/3 and FQ0 at the mature stage was obviously higher than that of the other treatments, whereas the abundance of fungal pathogens among each group in the root system did not change significantly (Figure 3).

### Relationship between microbial functional groups and N nutrition

**Figure 4:**
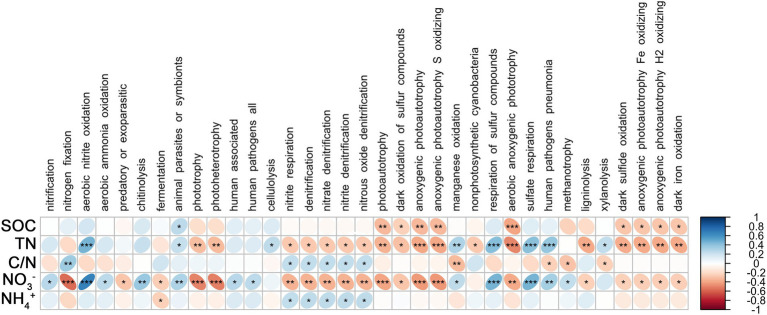
Correlations between predicted bacterial functional groups of the root and soil organic carbon (SOC), total nitrogen (TN), C/N ratio (C/N), soil nitrate nitrogen (NO_3_^−^), and ammonia nitrogen (NH_4_^+^) by Spearman’s rank correlation. Significant difference were indicated as follows: *p < 0.05; **p < 0.01; ***p < 0.001.

**Figure 5:**
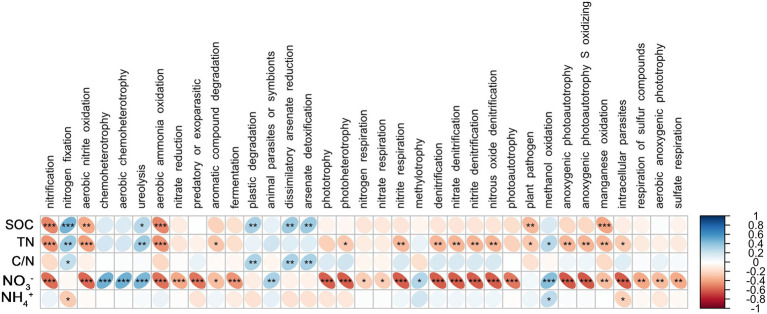
Correlations between predicted bacterial functional groups of the rhizosphere and soil organic carbon (SOC), total nitrogen (TN), C/N ratio (C/N), soil nitrate nitrogen (NO_3_^−^), and ammonia nitrogen (NH_4_^+^) levels based on Spearman’s rank correlation. Significant difference were indicated as follows: *p < 0.05; **p < 0.01; ***p < 0.001.

Spearman’s coefficient shows the correlation between bacterial functional groups and SOC, TN, C/N, NO_3_^−^, and NH_4_^+^. There was a significant positive correlation between root endophytic nitrification groups and NO_3_^−^ (*p* < 0.05); nitrogen-fixing groups were significantly and positively correlated with C/N (*p* < 0.01) and negatively correlated with NO_3_^−^ (*p* < 0.001), whereas the anaerobic nitrate-oxidising groups were significantly positively correlated with TN and NO_3_^−^ (*p* < 0.001; Figure 4). The nitrification groups in the rhizosphere soil were significantly negatively correlated with NO_3_^−^, SOC, and TN (*p* < 0.001), nitrogen-fixing group was positively correlated with SOC (*p* < 0.001), TN (*p* < 0.01), and C/N but negatively correlated with NH_4_^+^, and the anaerobic nitrate-oxidising groups were significantly negatively correlated with SOC, TN, and NO_3_^−^ (*p* < 0.01; Figure 5).

**Figure 6:**
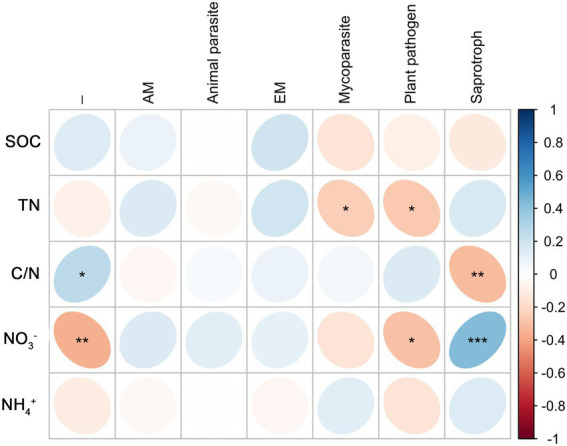
Correlations between predicted fungal functional groups of root and soil organic carbon (SOC), total nitrogen (TN), C/N ratio (C/N), soil nitrate nitrogen (NO_3_^−^), and ammonia nitrogen (NH_4_^+^) based on Spearman’s rank correlation. Significant difference were indicated as follows: *p < 0.05; **p < 0.01; ***p < 0.001.

**Figure 7:**
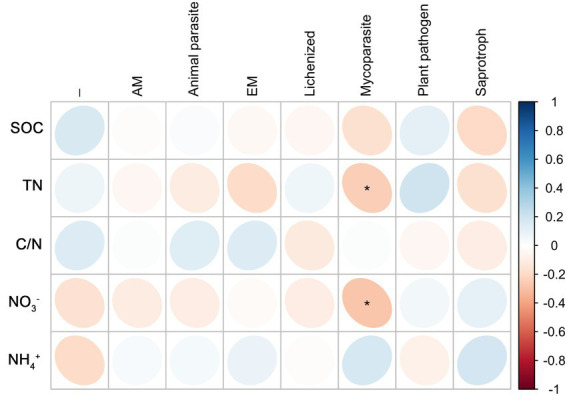
Correlations between predicted fungal functional groups of the rhizosphere and soil organic carbon (SOC), total nitrogen (TN), C/N ratio (C/N), soil nitrate nitrogen (NO_3_^−^), and ammonia nitrogen (NH_4_^+^) levels based on Spearman’s rank correlation. Significant difference was indicated as follow: *p < 0.05.

Root endophytic mycoparasite fungi were significantly positively correlated with TN; Fungal endophytic plant pathogens were significantly negatively correlated with TN and NO_3_^−^; a significantly negative correlation existed between Saprophytic fungi with C/N, and a significantly positive correlation with NO_3_^−^ (Figure 6). In rhizosphere soil, there was a significantly negative correlation between mycoparasite and TN and NO_3_^−^, whereas others were not correlated (Figure 7).

## Discussion

### Developmental stages and the functional guilds of microbiome

Previous studies have shown that the developmental stage of the crop profoundly affects the assembly and function of the plant microbiome, with bacterial and fungal microbiomes exerting different ecological roles at different stages (Xiong et al., 2020, 2021). In the early stage, the beneficial bacterial groups were more abundant in the plant microbiome, and the functional genes related to nutrient supply were enriched, rendering it the most important stage for nutrient enrichment (Xiong et al., 2021). In the late stage, more saprophytic and pathogenic fungi were enriched, and the processes of nitrogen assimilation and carbon degradation became dominant (Xiong et al., 2021). Thus, NO_3_^−^, TN, and SOC were mainly determined by the bacterial community at the jointing stage, NH_4_^+^ was mainly determined by the bacterial community at the flowering stage, and C/N was largely affected by the bacterial community at the mature stage. The effect of different tillage on the accumulation of N by root and rhizosphere fungal communities is continuous from sowing to flowering (Galazka and Grzadziel, 2018); therefore, the fungal community structure at the flowering and jointing stages affected NO_3_^−^ significantly.

Nitrification and anaerobic nitrate oxidation are two microbial biochemical reactions that cause major N losses in fertilised soil (Otaka et al., 2021). Mobile NO_3_^−^ produced by nitrification may leach out of the agricultural fields, polluting groundwater and affecting human health (Riuett et al., 2008; Ward et al., 2018). Moreover, the anaerobic nitrate-oxidising microbes reduce NO_3_^−^ to N_2_O or NO, two pollution and greenhouse gases that are 300 times more potent than CO_2_, which are then released from soil into the air (Lubbers et al., 2013; Hu et al., 2020). Therefore, agricultural management should be focused on inhibiting microbial nitrification and anaerobic nitrate oxidation driven by roots (Otaka et al., 2021). Conversely, nitrogen-fixing bacteria can convert N in the atmosphere into ammonia, which is conducive to crops (Batista and Dixon, 2019). Previous studies have shown that the frequency and amount of stover mulching can affect the microbial community, but it is not clear how they affect the bacteria related to the nitrogen cycle (Kou et al., 2020; Wang et al., 2020b). This study showed that the frequency of stover mulching, but not the amount, benefits the root and rhizosphere soil bacterial communities. Furthermore, we showed that full or no stover mulching does not inhibit nitrification and anaerobic nitrate oxidation, nor does it enrich nitrogen-fixing bacteria associated with roots. In contrast, stover mulching once every 3 years (F1/3) and twice every 3 years (F2/3) showed better results, yielding the lowest abundance of nitrifying and anaerobic nitrate-oxidising bacteria and the highest abundance of nitrogen-fixing bacteria.

The rhizo-microbiome usually shields plants from plant pathogens (Pollak and Cordero, 2020). The mature stage is the most important stage for soil health, because plant pathogenic fungi are more highly enriched at this stage than in other stages (Xiong et al., 2021). The abundance of plant pathogenic fungi in the rhizosphere under F2/3 treatment was the lowest of all functional groups, suggesting that the tillage twice every 3 years (F2/3) will inhibit the accumulation of plant pathogens.

### Associations between the functional guilds and soil nitrogen

The effects of nitrifying and anaerobic nitrate-oxidising bacteria in the roots and rhizosphere on soil N nutrition were inconsistent. The reason for this phenomenon is probably the competition between roots and soil microorganisms for N (Kuzyakov and Xu, 2013). Root endophytic microorganisms, as part of the root system, usually play the role of producers and have a different function from that of soil microorganisms. Nitrifying bacteria in roots always supply available NO_3_^−^ to plants and other microorganisms, whereas those in soil mainly cause N losses (Batista and Dixon, 2019; Otaka et al., 2021). Aerobic nitrite-oxidising bacteria in roots can help plants to obtain the essential Fe, but the same bacteria in soil can reduce NO_3_^−^ to N_2_O, thereby causing loss of N (Gu et al., 2020; Xue et al., 2020). Nitrogen-fixing bacteria, regardless of whether they are in the root or in the rhizosphere, are positively correlated with C/N. This is because stover is an organic matter with a high C/N ratio, and its decomposition aggravates N limitation in the rhizosphere, which stimulates nitrogen-fixing bacteria to fix more N (Kuzyakov and Xu, 2013; Oldroyd and Leyser, 2020; Li et al., 2021). During this process, the nitrogen-fixing bacteria increase SOC and N reserves, but they also consume available N (Song et al., 2020; Trivedi et al., 2020). Thus, nitrogen fixation by rhizobacteria was positively correlated with SOC, TN, and C/N, whereas the nitrogen-fixing bacteria in the root and rhizosphere were negatively correlated with NO_3_^−^ and NH_4_^+^, respectively.

In roots, autotrophic (such as nitrogen-fixing bacteria) and heterotrophic (such as saprotroph fungi) microorganisms usually inhibit each other (Kuzyakov and Xu, 2013; Oldroyd and Leyser, 2020; Xiong et al., 2021). In the present study, the saprotrophic fungi were negatively correlated with C/N and significantly positively correlated with NO_3_^−^, which was the opposite of the trend observed for nitrogen-fixing bacteria. Nitrogen nutrition strengthens the protection of roots and rhizosphere (Pollak and Cordero, 2020; Trivedi et al., 2020; Xue et al., 2020); therefore, plant pathogens and mycoparasitic fungi in roots were negatively correlated with TN, and the mycoparasitic fungi in the rhizosphere were negatively correlated with TN and NO_3_^−^.

## Conclusion

In this study, the microbial functional guilds in the root and rhizosphere were influenced by the frequency and amount of stover mulching. The bacterial functional guilds related to nitrogen fixation, nitrification, and anaerobic nitrate oxidation displayed the significantly correlation with C/N, TN, NO_3_^−^, and NH_4_^+^. The fungal functional guilds of plant pathogens and saprotrophs showed significantly correlations with C/N, TN, and NO_3_^−^. Moreover, we found that the ecological roles of bacteria and fungi significantly changed in different growth stages of crops, where nitrogen fixation, nitrification, and anaerobic nitrate oxidation play a pivotal role in maintaining N requirements at the jointing stage, whereas root endophytic fungi of plant pathogens and saprotrophs have an increasing impact on plant health at the mature stage. F1/3 and F2/3 showed the lowest abundance of nitrifying and anaerobic nitrate-oxidising bacteria, and the highest abundance of nitrogen-fixing bacteria at the jointing stage. F2/3 showed the lowest abundance of fungal plant pathogens in the roots at the mature stage. Together, these results suggest that F2/3 is the optimal stover mulching frequency to maintain plant health and N requirements in soil.

## Data availability statement

The datasets presented in this study can be found in online repositories. The names of the repository/repositories and accession number(s) can be found at: https://www.ncbi.nlm.nih.gov/, PRJNA758631.

## Author contributions

WS developed the ideas, designed the experimental plans, performed the experiments and wrote the manuscript. JW and LH analysed the data and drew the figures. All authors contributed to the article and approved the submitted version.

## Funding

This study was sponsored by the Key Laboratory of Ecology and Environment in Minority Areas (Minzu University of China), National Ethnic Affairs Commission (10301-2021000302) and Fundamental Research Funds for the Central Universities (10301-2021000301).

## Conflict of interest

The authors declare that the research was conducted in the absence of any commercial or financial relationships that could be construed as a potential conflict of interest.

## Publisher’s note

All claims expressed in this article are solely those of the authors and do not necessarily represent those of their affiliated organizations, or those of the publisher, the editors and the reviewers. Any product that may be evaluated in this article, or claim that may be made by its manufacturer, is not guaranteed or endorsed by the publisher.

## Supplementary material

SUPPLEMENTARY TABLE S1 The bacterial (Sheet1) and fungal (Sheet2) functional guilds in the samplings.
